# Do advance directive attitudes and perceived susceptibility and end-of-life life-sustaining treatment preferences between patients with heart failure and cancer differ?

**DOI:** 10.1371/journal.pone.0238567

**Published:** 2020-09-08

**Authors:** JinShil Kim, Jiin Choi, Mi-Seung Shin, Miyeong Kim, EunJu Seo, Minjeong An, Jae Lan Shim, Seongkum Heo

**Affiliations:** 1 College of Nursing, Gachon University, Incheon, South Korea; 2 Office of Hospital Information, Seoul National University Hospital, Seoul, South Korea; 3 Division of Cardiology, Department of Internal Medicine, Gil Medical Center, College of Medicine, Gachon University, Incheon, South Korea; 4 Gil Medical Center, Gachon University, Incheon, South Korea; 5 Department of Nursing, National Cancer Center, Seoul, South Korea; 6 College of Nursing, Chonnam National University, Gwangju, South Korea; 7 Department of Nursing, College of Medicine, Dongguk University, Gyeongju, South Korea; 8 Georgia Baptist College of Nursing, Mercer University, Atlanta, Georgia, United States of America; Maastricht University Medical Center, NETHERLANDS

## Abstract

There is limited evidence on the relationships of preference for end-of-life life-sustaining treatments [LSTs] and diagnostic contexts like heart failure [HF] or cancer, and patient attitudes toward and perceived susceptibility to use advance directives [ADs]. Thus, this study aimed to compare attitudes and perceived susceptibility between HF patients and community-dwelling patients with cancer, and examine the associations of these variables with their preference for each LST (cardiopulmonary resuscitation [CPR], ventilation support, hemodialysis, and hospice care). Secondary data were obtained from 36 outpatients with HF (mean age, 65.44 years; male, 69.4%) and 107 cancer patients (mean age, 67.39 years; male, 32.7%). More patients with HF preferred CPR than cancer patients (41.7% and 15.9%, *χ*^*2*^ = 8.88, *P* = 0.003). Attitudes and perceived susceptibility were similar between the two diagnostic cohorts. HF patients and those with more positive attitudes had greater odds of preferring CPR (odds ratio [OR] = 3.02, confidence interval [CI] = 1.19, 7.70) and hospice care (OR = 1.14, CI = 1.06, 1.23), respectively. HF diagnosis and AD attitudes increased the preference for CPR and hospice care, respectively. This suggests that it is important to gain positive attitudes toward ADs and consider diagnostic context to facilitate informed decision-making for LSTs.

## Introduction

Advances in medical therapeutics and management have prolonged patients’ survival rates after the diagnoses of heart failure (HF) [1], a non-cancerous disease but one of the most overwhelming progressive diseases [2, 3], and cancer [4, 5]. However, during the course of therapeutic treatment of illnesses, the burdens of care become substantial, often accompanying high morbidities and mortalities due to the chronic progressive nature of HF [1, 6], and cancer itself, treatment, or comorbid conditions [7–9]. The subsequent burdens in both diagnostic cohorts seem comparably high [10–13]. The burdens felt among patients with HF sometimes exceed those felt by patients with certain types of common cancers, such as prostate, breast, or bladder [11]. Actually, number of patients with HF among hospice care recipients after admission to a nursing home was twice that of cancer patients [10].Therefore, needs for advance care planning (ACP) in patients with HF and patients with advanced cancer were comparably high [13].

ACP is a systematic and comprehensive approach that could reduce the burdens of care in both diagnostic cohorts. This approach enables patients to actively engage in one’s therapeutic care through the process of ongoing communication with their healthcare providers and families to discuss goals of care and preferred medical care at the end-of-life (EoL) [14]. Before their condition becomes inevitably worse, patients can communicate with their healthcare providers and family for their preferences for life-sustaining treatments (LSTs) at the EoL on an advance directive (AD) and can review these preferences periodically [14–17]. Improved quality of care through ACP and/or ADs and the positive outcomes were documented in both diagnostic cohorts [18–22], including the reduced use of aggressive treatment near death [21, 22]. Nonetheless, late access to or lack of ACP and/or ADs often occurred in diagnostic contexts of both HF [20, 21] and cancer [23]. Limited access to or lack of ACP or ADs often leads patients with HF [21] and even those with advanced cancer [23] to receive aggressive treatments or care against their wishes near death. Access to ACP or ADs was less likely in patients with non-cancer than in patients with cancer [12, 24]. Lack of access to ACP and ADs can make patients with non-cancer diseases, including HF, receive less ACP and/or AD utilization and/or symptom management and comfort care than patients with cancer [12, 24, 25], and receive more aggressive treatments near death [25].

Based on several prior studies that reported substantial burdens of care [10, 11, 13] and demands for future EoL care planning [12, 13], but less access to ACP in patients with HF [12, 24] compared to the cancer population [12], this study hypothesized that diagnostic contexts (non-cancer HF vs. cancer) would be associated with consideration of an AD with primary decision-making for LST options, such as cardiopulmonary resuscitation (CPR) [10, 24]. In addition, such a decision may be influenced by some modifying factors, such as attitudes toward ADs [26, 27] and perceived susceptibility to AD use [28, 29]. We also hypothesize that a person with more positive attitudes and greater perceived importance for having unexpected EoL experiences without an AD is more likely to consider an AD. However, it remains uncertain how these factors play-out in patients with non-cancer HF and community-dwelling patients with cancer. Comparative insights about LST preferences between the two diagnostic cohorts (HF and cancer) will be informative to facilitate AD utilization and aid informed decision-making for LSTs based on their preferences, considering diagnostic contexts.

Thus, this study aimed to 1) compare preferences for LSTs (cardiopulmonary resuscitation [CPR], ventilation support, hemodialysis, and hospice care) between the two diagnostic cohorts, 2) compare AD attitudes and perceived susceptibility, and 3) examine associations of the diagnosis contexts, and AD attitudes and perceived susceptibility with each of the four LST preferences.

## Materials and methods

### Design and procedure

This study used a secondary data analysis design. Part of the data on LSTs and attitudes toward ADs and perceived susceptibility to use ADs were obtained from patients with HF and community-dwelling patients with cancer. The preferences for LSTs between the two groups were compared, and the associations of attitudes, perceived susceptibility, and diagnostic context with preferences for LSTs were assessed. The institutional review board of Gachon University Gil Medical Center approved this secondary analysis (Ethical code: GCIRB2020-156).

The original research findings are published elsewhere [30, 31]. In a study with patients with HF and their caregivers who were recruited at the outpatient of the university-affiliated hospital, dyadic agreement for LSTs and correlates of each LSTs were examined. In a study with community-dwelling patients with cancer who were recruited from a local public health center, decisional conflicts associated with each LSTs were examined. They were the recipients of a nurse-visiting cancer management service which was designed for assisting low-income, home-based cancer survivors with cancer survivorship issues. Both groups of patients with HF and patients with cancer provided written informed consent before data collection at outpatient visits for routine care and during the nurses’ home visits for cancer management assistance, respectively.

### Participants

The eligibility criteria for patients with HF were those who (1) were aware of their HF diagnosis and were receiving optimal pharmacologic treatments, such as beta-blockers, angiotensin-converting-enzyme inhibitors, or angiotensin receptor blockers, and (2) completed the AD survey questionnaire, including the LST preferences. Patients with cancer who were qualified for home visits for cancer management, such as those who were financially vulnerable and within the bottom quintile of the minimum cost of living or were vulnerable regarding health insurance payments as well as those who were diagnosed and completed cancer treatment in less than five years [32], were included in this study. For both populations, exclusion criteria were terminally ill patients who met palliative and hospice care candidacy, such as those with terminal cancer, chronic obstructive pulmonary disease, liver cirrhosis, or acquired immune deficiency syndrome. Those with a documented neurological or mental disorder, such as dementia, Alzheimer’s disease, or brain or mental disorders which restrict individual autonomy for medical decision-making were also excluded.

### Measures

#### LST preferences

Preferences for future LSTs were assessed using part of the Korean-Advance Directive (K-AD) questionnaire that consists of EoL values as well as desired LSTs and surrogate decision-makers for medical care [33]. Assuming an EoL moment, patients were given four LST options (CPR, ventilation support, hemodialysis, and hospice care) for non-cancer populations and an additional option of chemotherapy for the cancer population. Research subjects received minimum explanation about the basic meaning of each LST and were asked to indicate their preference on a dichotomous scale (1 = yes; 0 = no). In prior studies, the K-AD model has been administered to various populations, including patients with HF and cancer and their caregivers in clinical practice [31, 33], community-dwelling elderly people, and community-dwelling patients with cancer [30, 33].

#### Attitudes toward Ads

Attitudes were assessed using the 16-item Advance Directive Attitude Survey (ADAS) [34]. The extent of an individual’s positive-negative attitudes for ADs was assessed on a four-point Likert scale (1 = strongly disagree; 4 = strongly agree) for each item in four sections. The possible scores ranged from 16 to 64, with a higher score indicating greater positive attitudes toward ADs. Reliability was supported, with Cronbach’s alphas of 0.74 for the original questionnaire [34] and 0.80 for the Korean version [35].

#### Perceived susceptibility

Perceived susceptibility was assessed using the 5-item Perceived Susceptibility subscale, a part of the Advance Care Planning survey [29]. Each item captures individuals’ perceived importance for having unwanted EoL experiences when one does not document desired care at the EoL on an AD, and is rated on a seven-point Likert scale (1 = absolutely not important; 7 = very important). The possible scores ranged from 5 to 35, with a higher score indicating greater perceived importance for experiencing unwanted EoL events. In a previous study, reliability was supported, with a Cronbach’s alpha of 0.73 among older adults [29].

#### Demographic and clinical characteristics

Using a standard form, both patient populations provided demographic information including age, sex, marital status, employment status, caregiver attendance, and educational level. For patients with HF, functional severity of HF was assessed using the New York Heart Association classification system, in which patients were classified from asymptomatic class I to severely symptomatic and functionally limited class IV based on HF symptom entailed functional limitation. Electronic medical records were also reviewed to collect clinical information including duration of illness, etiology, left ventricular ejection fraction, and prescribed medications. Community-dwelling patients with cancer self-reported time since cancer diagnosis and type of cancer.

### Statistical analysis

Descriptive statistics were used to describe the sample characteristics and included central tendency (mean and standard deviation; median and 1st quartile (Q1), 3rd quartile (Q3), and frequency and percentage. Chi-square tests or *t*-tests were conducted to compare sample characteristics between patients with HF and cancer. Wilcoxon rank sum test and multivariable logistic regression analyses were also performed. Bivariate comparisons were performed to compare preferences for LSTs between the two diagnostic cohorts, and attitudes toward ADs and perceived susceptibility of illness were assessed using *t*-test and Wilcoxon rank sum test. A series of multivariable logistic regression analyses, with computation of odds ratios (OR) and confidence intervals (CIs), were performed to examine and identify associations of each LST preference in which attitudes and perceived susceptibility, as well as diagnosis of HF with cancer as a reference, were entered as predictors, and sex, marital status, and educational level were entered as covariates, which showed significant group differences in this study. All statistical analyses were performed using the software package R version 3.6.0 [36]. The level of significance was set at p < 0.05.

## Results

A total of 36 patients with HF (mean age, 65.44 ± 12.85 years) and 107 patients with cancer (mean age, 67.39 ± 11.57 years) were included in this study (Table 1). There were more males in the HF cohort than cancer cohort (69.4% vs. 32.7%, *p* < 0.001). More patients with HF (75.0%) than patients with cancer (51.4%) were married (*p* = 0.023). Education level in patients with HF was higher than that in patients with cancer (58.3% vs. 34.6%, *p* = 0.021). Only 16.7% of patients with HF and 30.8% of patients with cancer reported that they heard of the ADs (*p* = 0.151).

**Table 1:** Demographic and clinical characteristics of patients with heart failure and community-dwelling patients with cancer (N = 143).

Variables		Patients with HF (n = 36)	Patients with Cancer (n = 107)	*t or χ^2^ (P* value*)*
		n (%) or Mean ± SD		
Age, years		65.44 ± 12.85	67.39 ± 11.57	0.85 (0.397)
Sex	Male	25 (69.4)	35 (32.7)	13.5 (<0.001)
Marital status	Married	27 (75.0)	55 (51.4)	5.21 (0.023)
Occupation	Yes	11 (30.6)	18 (16.8)	2.35 (0.125)
Caregiver	Yes	25 (69.4)	65 (60.7)	0.54 (0.462)
Education	< high school	15 (41.7)	70 (65.4)	5.36 (0.021)
	≥ high school	21 (58.3)	37 (34.6)	
HF duration, months*		36.0 (9.0, 102.5)		
Left ventricular ejection fraction, %		36.58 ± 9.91		
NYHA classes	I	7 (19.4)		
	II	21 (58.3)		
	III	8 (22.2)		
	IV	0 (0.0)		
Ischemic etiology		22 (61.1)		
Medication, yes	ACEI	21 (58.3)		
	ARB	11 (30.6)		
	Beta-blockers	32 (88.9)		
	Loop diuretics**	17 (47.2)		
Cancer duration	> 5 years		52 (48.6)	
Cancer origin	Gastric / colon		40 (37.4)	
	Breast		21 (19.6)	
	Lung		7 (6.5)	
	Liver		6 (5.6)	
	Metastasis		6 (5.6)	
	Ovary/Uterus/Cervix	5 (4.7)	
	Pancreas / Biliary		3 (2.8)	
	Others		19 (17.8)	
Awareness of an AD (yes)		6 (16.7)	33 (30.8)	2.06 (0.151)

Abbreviations: ACEI, angiotensin-converting enzyme inhibitor; AD, advance directives; ARB, angiotensin receptor blocker; HF, heart failure; NYHA, New York Heart Association; SD, standard deviation.

* Presented by 1^st^ quartile and 3^rd^ quartile (Q1, Q3).

** Includes furosemide or torsemide.

Note. Normally and non-normally distributed variables were presented by mean ± SD and median (Q1, Q3), respectively.

For patients with HF, the median time after HF diagnosis was 36.0 months (Q1 = 9.0, Q3 = 102.5). The majority of patients with HF (77.7%) were at NYHA class I or II. Most patients with cancer had solid cancer in the gastrointestinal system (37.4%), breast (19.6%), and liver/pancreas/biliary duct (8.4%). Less than half were those with a cancer duration after diagnosis greater than 5 years (48.6%).

### Comparison of LST preferences between patients with heart failure and patients with cancer

In patients with HF, preference for aggressive treatments ranged from 27.8% for ventilation support and hemodialysis to 41.7% for CPR, while preference for hospice care was 69.4% (Table 2). In patients with cancer, preference for aggressive treatments ranged from 15.0% for ventilation support to 18.7% for hemodialysis, while preference for hospice care was 66.4%. In associational analyses between diagnostic context and each of the LSTs, only the association between diagnostic context and CPR was significant. More patients with HF compared with those with cancer had a higher preference for CPR (41.7% and 15.9%, *χ*^*2*^ = 8.88, *p* = 0.003).

**Table 2:** Comparisons of preferred life-sustaining treatments between patients with heart failure and cancer (N = 143).

Treatment wishes		Patients with HF (n = 36)	Patients with Cancer (n = 107)	*χ^2^ (P* value*)*
		n (%)	n (%)	
Cardiopulmonary resuscitation	Yes	15 (41.7)	17 (15.9)	8.88 (0.003)
	No	21 (58.3)	90 (84.1)	
Ventilator support	Yes	10 (27.8)	16 (15.0)	2.18 (0.140)
	No	26 (72.2)	91 (85.0)	
Hemodialysis	Yes	10 (27.8)	20 (18.7)	0.85 (0.357)
	No	26 (72.2)	87 (81.3)	
Hospice care	Yes	25 (69.4)	71 (66.4)	0.02 (0.892)
	No	11 (30.6)	36 (33.6)	

Abbreviation: HF, heart failure.

### Comparison of advance directive attitudes and perceived susceptibility between patients with heart failure and patients with cancer

Both groups of patients with HF and patients with cancer demonstrated similarly high positive attitudes toward ADs (46.81 and 47.75, respectively; *p* = 0.508). The level of perceived susceptibility to having unwanted EoL experiences was also similar between the two diagnostic cohorts (*p* = 0.062) (Table 3).

**Table 3:** Comparisons of advance directive attitudes and perceived susceptibility between patients with heart failure and cancer patients (N = 143).

Characteristics	Patients with HF (n = 36)	Patients with Cancer (n = 107)	*t (P* value*)*
	Mean ± SD or median (Q1, Q3)		
AD attitudes	46.81 ± 7.74	47.75 ± 6.00	0.67 (0.508)
Perceived susceptibility	23 (18, 29)	26 (20, 32)	0.062*

Abbreviations: AD, advance directives; HF, heart failure; SD, standard deviation.

* Wilcoxon *P* value.

Note. Normally distributed variables were presented by mean ± SD. Non-normally distributed variables were presented by median (Q1, Q3).

### Relationships of diagnostic contexts, advance directive attitudes, and perceived susceptibility to each LST preference

A series of multivariable logistic analyses were conducted to examine the associations of diagnostic contexts, AD attitudes, and perceived susceptibility with each LST preference. Sex, marital status, and educational level were entered into the models as covariates and each of the four LST preferences as a dependent variable in separate models (Table 4). HF diagnosis was associated with a greater likelihood of preferring CPR (odds ratio [OR] = 3.02, 95% confidence interval [CI] = 1.19, 7.70); the odds of preferring CPR in patients with HF were almost three times higher than that in patients with cancer. Advance directive attitudes were associated with a greater likelihood of preferring hospice care (OR = 1.14, 95% CI = 1.06, 1.23); a one-point increase in the AD attitudes score (more positive attitudes) resulted in a 14% increase in the preference for hospice care. Males were more likely to prefer hemodialysis than females (OR = 4.57, 95% CI = 1.78, 11.71). The overall goodness of fit based on the Chi-square test for each model was appropriate, except for ventilation support.

**Table 4:** Relationships of the diagnostic context, attitudes, and perceived susceptibility with each life-sustaining treatment preference.

Outcome variable	Factor^a^	B	*P* value	Exp (B)	95% CI	
					Lower	Upper
Cardiopulmonary resuscitation	Male	0.65	0.160	1.92	0.77	4.75
	Married	0.25	0.610	1.28	0.50	3.29
	High school education	-0.32	0.493	0.72	0.29	1.83
	HF diagnosis	1.10	0.021	3.02	1.19	7.70
	Attitudes	-0.06	0.089	0.94	0.88	1.01
	Perceived susceptibility	-0.01	0.726	0.99	0.94	1.04
		Model test: Chi-square = 15.51, *P* = 0.017				
Ventilation support	Male	0.92	0.060	2.52	0.96	6.62
	Married	0.003	0.995	1.00	0.38	2.68
	High school education	-0.28	0.577	0.76	0.29	2.00
	HF diagnosis	0.50	0.326	1.65	0.61	4.52
	Attitudes	-0.05	0.163	0.95	0.88	1.02
	Perceived susceptibility	-0.01	0.807	0.99	0.94	1.05
		Model test: Chi-square = 8.91, *P* = 0.179				
Hemodialysis	Male	1.52	0.002	4.57	1.78	11.71
	Married	-0.02	0.968	0.98	0.39	2.47
	High school education	-0.33	0.486	0.72	0.29	1.80
	HF diagnosis	0.15	0.762	1.17	0.43	3.17
	Attitudes	0.002	0.979	1.002	0.93	1.07
	Perceived susceptibility	0.02	0.511	1.02	0.96	1.08
		Model test: Chi-square = 13.18, *P* = 0.040				
Hospice care	Male	0.46	0.283	1.58	0.69	3.63
	Married	-0.01	0.984	0.99	0.42	2.31
	High school education	0.10	0.825	1.10	0.47	2.57
	HF diagnosis	0.24	0.623	1.28	0.48	3.36
	Attitudes	0.13	<0.001	1.14	1.06	1.23
	Perceived susceptibility	0.03	0.330	1.03	0.97	1.08
		Model test: Chi-square = 23.06, *P* = 0.001				

Abbreviations: CI, confidence interval; HF, heart failure.

^a^Each model includes sex (reference, female), marital status (reference, unmarried), education (reference, less than high school), heart failure diagnosis (reference, cancer), attitudes, and perceived susceptibility.

## Discussion

This study initially compared each LST preference, AD attitudes, and perceived susceptibility between patients with HF and cancer, and the associations of diagnostic contexts, AD attitudes, and perceived susceptibility with each of LST preferences in South Korea. There were no differences in AD attitudes and perceived susceptibility between patients with HF and cancer. However, diagnostic contexts and AD attitudes were associated with preference for CPR and hospice care, respectively. The likelihood of preferring CPR increased with HF diagnosis, while the likelihood of preferring hospice care was increased with increase in positive attitudes. These findings indicate that improving AD attitudes can be an important target of interventions to facilitate informed decisions about LSTs, particularly hospice care in both patients with HF and patients with cancer. In addition, the findings of this study also indicate that clinicians and researchers need to consider diagnostic contexts in AD-related counseling and discussion.

Integration of ACP and/or use of ADs into routine management of HF is highly recommended, particularly for those who at the EoL stage, due to the progressive deterioration of HF [37]. In South Korea, such type of care in HF has recently received attention, and experts in cardiovascular care have reached a consensus to initiate ACP communication to facilitate informed decision-making for future EoL care [38]. More aggressive care [25] and less comfort care [12, 24, 25] at the EoL are more likely to occur without having ACP process or ADs. In one study, EoL care in patients with HF was more aggressive than that in patients with cancer [25]. Thus, it is important to examine differences in preferences for LST wishes and factors associated with LST wishes depending on diagnostic contexts of HF and cancer. We found that LST wishes were similar between patients with HF and patients with cancer, except for CPR with patients with HF having a more favor (41.7%) than cancer patients (15.9%). These findings are consistent with those in prior studies. For example, 79.5% of outpatients with cancer who had mostly solid tumors preferred hospice care, while only approximately 20% preferred CPR and ventilation support [33]. In chronically ill older adults, the patterns were similar (54.5% preferring hospice care, while 14.3% preferring CPR) [39]. These findings in the current and prior studies imply that aggressive treatments, particularly CPR, were more highly preferred in patients with HF compared to outpatients with cancer, community-dwelling patients with cancer, and older people with chronic diseases. Previously, limited information about EoL treatments led terminally ill patients to choose CPR [40]. The findings of this study show that diagnostic context may affect choosing CPR. Thus, it is important to consider the diagnostic contexts and healthcare settings are required in the discussion about EoL medical care, including decisions for LSTs.

We further examined whether diagnostic contexts affected the decision-making for LSTs because having a severe medical diagnosis was one of the significant factors for considering ACP discussions in community-dwelling individuals in a prior study [41]. We investigated whether diagnoses of HF and cancer were associated with EoL treatment preferences. Diagnosis of HF was associated only with the odds of preferring CPR, with its odds increasing approximately three times than that associated with the diagnosis of cancer. Among patients with non-cancer compared to those with cancer, intensive and invasive care and LSTs at the EoL were more common and often initiated near death, whereas symptom management and comfort care were not offered extensively [25, 42]. This may, in part, explain the finding of this study that shows more preference for CPR among HF patients without a cancer diagnosis compared to patients with cancer. Patients with HF compared to patients with cancer might believe their conditions to be less serious and curable, leading to more preference for CPR until near EoL. However, both patients with HF and patients with cancer did not differ in preferences for hospice care, and hospice care was the most preferred LST. This may be related to the fact that people put the most value on peaceful deaths regardless diagnoses, which may lead their decision-making for comfort care options, such as hospice care, to be easier than those of aggressive life-prolonging options. These findings support the consideration of diagnostic contexts in ACP discussions to facilitate adequate discussion and decision-making regarding EoL care and LSTs.

In addition to the associational relationships of the diagnostic contexts with EoL treatment preferences, we also examined possible associations of AD attitudes and perceived susceptibility with EoL treatment preferences because prior studies showed possible associations of AD attitudes and perceptions with an individual’s decisions for the desired EoL treatments [26–29, 43]. However, little is known about these links. More positive attitudes were associated with higher likelihood of hospice care preference only. AD attitudes as a factor associated with the ACP discussion and/or AD documentation were supported in the limited number of prior studies. Among the hematopoietic cell transplant candidates, those with more positive attitudes toward ADs were more likely to complete ADs [43]. In one study that investigated the effects of ACP intervention in decompensated patients with HF, baseline positive AD attitudes did not significantly change after the intervention and were not associated with the use of AD [26]. While in another pilot study that tested ACP intervention for low-income older adults, attitudes toward ADs significantly increased after intervention [44]. The findings of this and previous studies showed possible roles of AD attitudes in LST wishes, or ADs, but further studies are needed to examine the causal relationships more thoroughly and develop and deliver more effective interventions to improve ACP and/or use of ADs.

In addition, among the non-modifying demographic factors, men were associated with a higher preference for aggressive treatment of hemodialysis in this study. The findings regarding the role of one’s sex in ACP discussions and/or ADs in previous studies were inconsistent. However, prior research shows that men were significantly associated with more documented ACP discussions among community-dwelling older adults [45], whereas females were associated more with AD completion among the hospitalized and community-dwelling older adults [46]. One possible reason for the inconsistent findings in our study and the prior study may be differences in the outcomes; we examined the associations between sex and specific LST preferences, while prior studies examined the associations between sex and ACP or AD documentation. Thus, further studies are needed to examine further relationships of sex to each LST preference, ACT, and AD documentation.

### Study strengths and limitations

This study was the first to report that diagnostic contexts of HF and cancer and AD attitudes are associated with EoL treatment preferences. This study also provided important insights into AD perspectives among patients with HF whose AD attitudes and illness perceptions were comparable to those of patients with cancer. However, this study had some limitations. Secondary analysis study design is a major limitation of this study. Secondary analysis can prevent precisely capturing the phenomenon of interest, which examined LST preferences associated with the diagnostic contexts and other potential influential factors. Such a design also restricts making conclusive relationships; thus, it is uncertain whether comparable results regarding EoL treatment preferences between the two diagnostic cohorts, except for CPR, reflected the diagnostic contexts or were yielded by other influences. The 2018 execution and enforcement of the Act on Life-Sustaining Treatment Determination [47] and the nationwide campaign regarding its benefits, AD registration and non-cancer population’s increased recognition could have been influencing factors. Another limitation is the difference in sample characteristics between patients with HF and those with cancer. Cancer patients came from low income groups, unlike patients with HF. This fact may impact the findings of this study because social class can impact preferences to LSTs [48]. Thus, the results of this study need to be further verified with a more rigorous study design and using financially equitable samples.

## Conclusions

In this study, aggressive treatments, particularly CPR, were more highly preferred among patients with HF than in patients with cancer. However, both had a similar preference for hospice care and the rest of aggressive treatments. Both diagnostic cohorts had highly positive AD attitudes; more positive attitudes increased the odds of preferring hospice care. Further, a diagnostic context of HF increased the odds of preferring CPR. Thus, enhancing attitudes and considering diagnostic contexts could facilitate informed decision-making for end-of-life treatments.

### Implications for practice and research

As the topic of ACP and ADs has recently received attention in East Asian countries [49], the results of this study have several implications in clinical practice and research. It seemed feasible to begin ACP discussions during the early stages of illness in patients with HF whose attitudes and perceived susceptibility were similar to those of patients with cancer in the community. Improvement in AD attitudes, consideration of diagnostic contexts, and periodic, ongoing discussions of LST wishes in primary and palliative supportive care may increase utilization of ACP and/or ADs and informed decision-making for future care in patients with HF and cancer.

The high preference for EoL aggressive treatments, particularly CPR in patients with HF, and the significant association of better attitudes with increased likelihood of preferring hospice care highlight what could be targeted when designing ACP interventions to avoid futile treatments and allow timely referrals to hospice care during EoL [50]. Further, it is necessary to develop malignant and non-malignant models that are appropriate for each diagnostic group. Further research is also warranted to investigate whether more factors (that we could not include) may impact preference to each EoL LST.

## Supporting information

S1 Dataset (ZIP)
